# Piloting Novel Nutritional Interventions for Mechanistic Studies of Human Aging

**DOI:** 10.1093/geroni/igaf122.1391

**Published:** 2025-12-31

**Authors:** Corby Martin, Leanne Redman

**Affiliations:** Pennington Biomedical Research Center; Pennington Biomedical Research Center, Baton Rouge, Louisiana, United States

## Abstract

Calorie restriction (CR) and time-restricted eating (TRE) have demonstrated efficacy for improving healthspan and biological age, yet long-term adherence remains problematic. Adaptive interventions may promote adherence in longer trials. This 24-week, parallel-arm, randomized controlled pilot trial compared adaptive and traditional versions of TRE and CR against a no-intervention control. Seventy (n = 70) healthy adults with body mass index 22.0-29.9 kg/m2 were randomized to one of five groups: control, traditional TRE with an 8-hour window, adaptive TRE, traditional CR targeting 25% energy restriction, or adaptive CR. The study aimed to examine intervention feasibility and effect sizes for primary aging and healthspan. Participants were 38±3 y (Mean+SD), 77% female, with mean BMI 26.0±2.6 kg/m2. The CR arms achieved 48% (traditional) and 77.6% (adaptive) of the CR target. TRE participants adhered to the 8-hour eating window on 74.5% (traditional) and 40.1% (adaptive) of days, respectively (86% adherence reflected the goal of following TRE 6 days per week). Weight change compared to control was greatest in adaptive CR (-8.8 kg, P < 0.001) followed by traditional CR (-5.8 kg, P < 0.001), traditional TRE (-2.3 kg, P = 0.05) and adaptive TRE (-1.4 kg, P = 0.15). Compared to control, biological age (Klemera-Doubal) effect sizes were modest over 24-weeks, with adaptive CR having the largest change (-1.8 y, P = 0.10; Cohen’s d = 0.24). Compared to control, healthspan (Metabolic Syndrome Score) improved in the CR arms (traditional -2.2, P < 0.01; adaptive -1.8, P = 0.02) but not in the TRE arms (traditional -0.6, P = 0.26; adaptive -0.7, P = 0.21). Intervention feasibility was highest in the adaptive groups.

